# Enhanced Distal Signaling in Human Hippocampal Neurons despite Lower Intrinsic Excitability

**DOI:** 10.21203/rs.3.rs-7820914/v1

**Published:** 2025-11-07

**Authors:** Tanvi Butola, Vincent Robert, Buyong Kim, Werner Doyle, Fabliha Hussain, Cheng Gong, Yoni Leibner, Olesia Bilash, Keelin O’Neil, Lulu Peng, Miranda Duster, Alia Seedat, Sasha Devore, Yuxiu Katherine Wang, Sarra Belakhoua, Christopher William, Daniel Friedman, Idan Segev, Raju Tomer, Orrin Devinsky, Jayeeta Basu

**Affiliations:** 1 New York University Langone Health; 2 Department of Neuroscience, Institute for Translational Neuroscience, New York University Grossman School of Medicine; 3 Department of Neurology, New York University Grossman School of Medicine; 4 Department of Neurosurgery, New York University Grossman School of Medicine; 5 Department of Pathology, New York University Grossman School of Medicine; 6 Department of Psychiatry, New York University Grossman School of Medicine; 7 Center for Neural Science, New York University; 8 Department of Biological Sciences, Columbia University; 9 Department of Biomedical Engineering, Columbia University; 10 The Edmond and Lily Safra Center for Brain Sciences, Hebrew University of Jerusalem, Israel

## Abstract

The hippocampus is critical for memory and spatial navigation, and is central to the pathophysiology of temporal lobe epilepsy — the most common drug-resistant epilepsy. Yet our understanding of the cortico-hippocampal circuit, and neuronal function relies on rodent studies, which may not fully model human features. Here, we combined patch-clamp electrophysiology, histology, and microscopy to functionally and morphologically characterize human hippocampal neuron types at high-resolution from freshly resected tissue from epilepsy patients. We found striking region-, species, and pathology-specific differences in neuronal excitability, synaptic dynamics, and dendritic branch patterns. Dentate gyrus granule cells are the most excitable human hippocampal principal neurons. Human neurons are intrinsically less excitable than mouse neurons—requiring more current to fire—but paradoxically show higher action potential firing rates. Human pyramidal neurons from non-sclerotic CA1 show reduced sag compared to their sclerotic tissue, and its mouse counterpart. Human neurons more effectively preserve distal dendritic signal propagation to the soma. Neurons in each hippocampal sub-region display distinct activity-dependent synaptic plasticity dynamics. Morphologically, human neurons are larger with more elaborate and diverse dendritic branching patterns. Taken together, our results suggest that human hippocampal principal neurons have evolved in form and function to enhance synaptic input integration, and signaling.

## Introduction

Our understanding of brain circuits and neuron types primarily comes from animal models. How valid is this extrapolation? Comparing human and other mammalian species, many neuronal types and circuits are conserved^1,2^, although accumulating evidence points to morphological, biophysical, and computational differences between rodent and human neurons^3–8^. These species-specific features raise questions about how faithfully animal models can capture human circuit function and predict therapeutic responses. Indeed, drugs developed from animal models have <12% success in human trials^9^. Thus, it is imperative to compare the structure and function of human neurons^10,11^ and circuits with those in animal models.

The issue of species-specific differences is particularly pressing to understand in the hippocampus, a structure central to episodic memory^12–17^ and spatial navigation^18–21^, and a major site of pathology in temporal lobe epilepsy (TLE)^22–25^, —the most common drug-resistant epilepsy^26^. While rodent studies have dominated our view of hippocampal function, differences in gene^27^, dendritic morphologies^7,28^, and dendritic spines^29–32^ between human and rodent pyramidal neurons suggest potentially distinct rules for excitability and computation. Although such differences can alter cable properties and transfer functions^28,33–38^, direct recordings of human neurons remain limited, leaving open fundamental questions about how these differences shape signal processing, synaptic connectivity^39^ and input dynamics, and non-linear computations such dendritic integration^40^ within human neuronal sub-populations. These factors are affected in mouse models of epilepsy but how they are affected and contribute to disease vulnerability in humans remains unknown^39–42^.

**Extended Data Figure 1:**
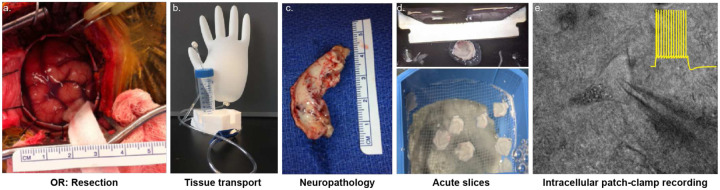
Workflow of obtaining resected human brain tissue for investigation a. Surgical site in the operating room (OR) before brain tissue resection. Cranial window 3 cm in diameter exposes the region of interest. b. Apparatus to transport resected human tissue. A large glove filled with carbogen (95% O_2_, 5% CO_2_) acts as an oxygen reservoir for the transport solution in the falcon tube. Resected human brain tissue transported in modified artificial cerebrospinal fluid with constant oxygenation ensures the health of the tissue during transport. c. The resected tissue is first taken to neuropathology to be examined for tumors. Once cleared, it is transported to the laboratory. d. In the laboratory, the tissue is acutely sliced in 400 μm thick sections and can be maintained in oxygenated artificial cerebrospinal fluid (aCSF) at room tem perature for up to 72 hours. e. Bright-field image of the field of view under the microscope while performing intracellular patch-clamp recordings from human neurons. The trace in yellow is a sample traces of action potential firing of a human neuron when injected with depolarizing current.

To fill this gap, we established a close collaboration between the Basu Lab, the Epilepsy Center, and several clinical departments at NYU Langone Health to study neurons, synapses, and circuits in human hippocampal sub-regions in brain tissue resected from 11 TLE patients undergoing surgical resection of the temporal neocortex and hippocampus. The proximity between the neurosurgery operating room, neuropathology screening, and the Basu Lab minimized the transit time for resected brain tissue within 9 minutes, while maintaining optimal oxygenation and tissue health. (Extended Figure 1). We prepared viable slices for high-quality^43,44^, single-cell intracellular recordings for up to 72 hrs post-resection from dentate gyrus (DG), CA3/CA2, and CA1 hippocampal sub-regions, coupled with synaptic stimulation of segregated input pathways.

**Figure 1:**
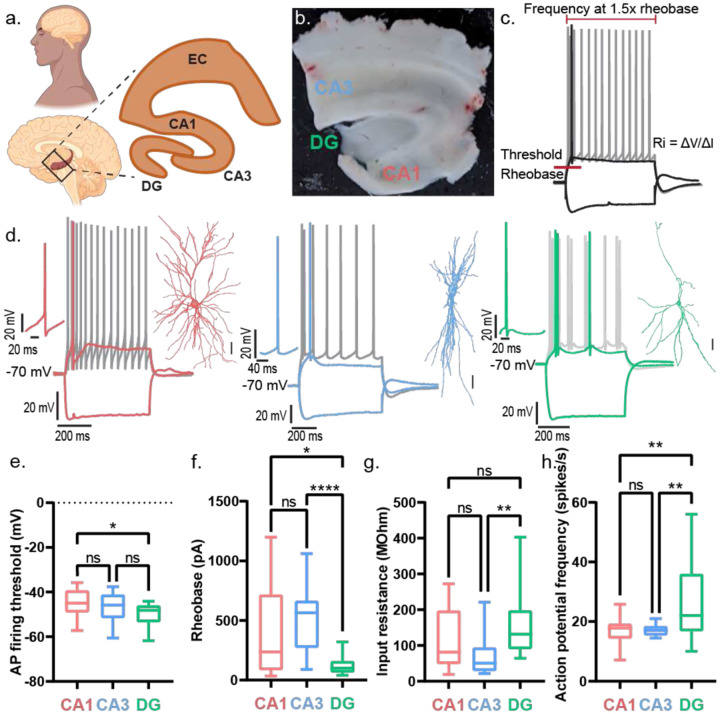
Human dentate gyrus demonstrates higher intrinsic excitability than hippocampal CA1 and CA3 in resected brain tissue. a. Schematic showing a sagittal section of a human brain with a box highlighting the hippocampus. Inset zooms in on the human hippocampus showing the different sub regions – dentate gyrus (DG), CA1, CA3, and entorhinal cortex (EC). b. Image shows a 400 μm thick acute section obtained from human brain tissue resected from epilepsy patients undergoing surgical treatment for refractory epilepsy. Different hippocampal sub regions (CA1, CA3, and DG) demarcated within the slice from where electrophysiological recordings were obtained. c. Sample traces of fire and sag properties from a hippocampal neuron in response to depolarizing and hyperpolarizing current injections respectively. Figure panel demonstrates the parameters measured to assess the intrinsic excitability of human hippocampal neurons: AP firing threshold, rheobase (minimum current required to elicit action potential (AP)), input resistance of the neuronal membrane (Ri) and AP frequency measured at depolarizing current injection of 1.5 times the rheobase. d. Representative traces of fire and sag properties recorded from human CA1 (red), CA3 (blue), and DG (green) in response to depolarizing and hyperpolarizing current injections respectively. Colored firing trace in each panel corresponds to AP firing at rheobase, while the gray firing trace corresponds to AP firing at current injection 1.5 times the rheobase. Left inset shows a magnified view of a single AP. Right inset shows a reconstructed neuron that was filled with dye during electrophysiological patch-clamp recordings and subsequently stained and imaged. Scale bar – 25 μm. Fire and sag properties, and neuron morphological helped confirm the neuronal type recorded. e-h. AP firing threshold (e), rheobase (f), input resistance (g), and action potential frequency (h) presented as box and whisker plots (median, lower/upper quartiles and min-max values). Statistical significance was tested with one-way ANOVA with Tukey’s post-hoc correction for multiple comparisons. * p-value <0.05, ** p-value <0.01, **** p-value <0.0001, ns – not significant.

**Figure 2:**
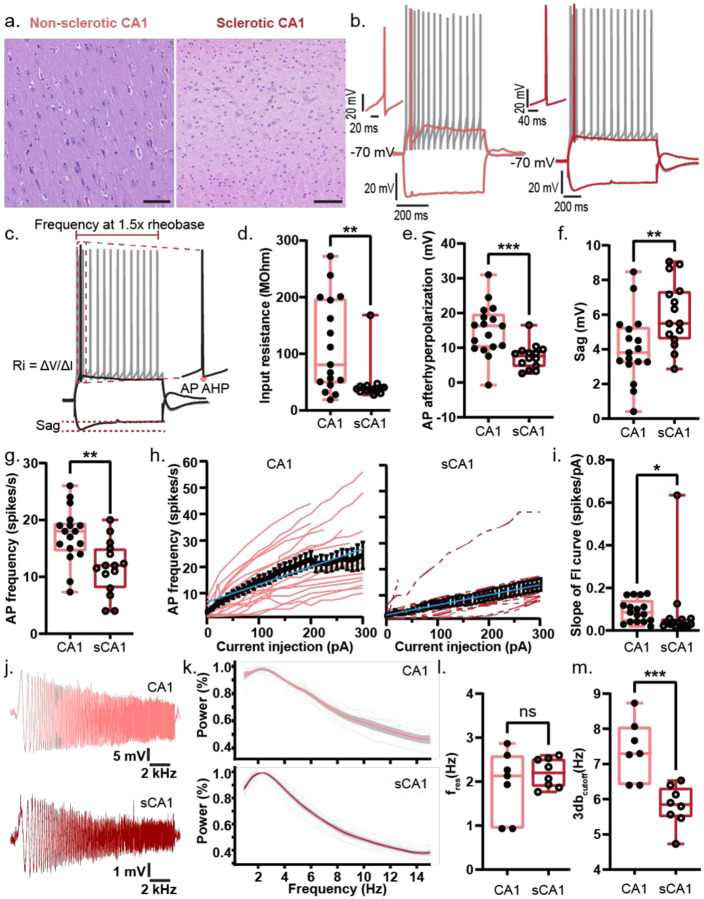
Reduced intrinsic excitability and altered HCN-mediated properties in sclerotic human CA1 neurons a. Hippocampal CA1 with hematoxylin-eosin stain highlighting normal neuronal architecture and population in non-sclerotic tissue (*left*) versus neuronal loss and aberrant cellular architecture in sclerotic tissue (*right*). Scale bar – 100 um. b. Representative traces of fire and sag properties recorded from human non-sclerotic (light red, left) and sclerotic (red, right) CA1 in response to depolarizing and hyperpolarizing current injections. Colored firing trace corresponds to AP firing at rheobase, while the gray firing trace corresponds to AP firing at current injection 1.5 times the rheobase. Left inset shows a magnified view of a single AP. c. Sample traces of fire and sag properties from a hippocampal neuron in response to depolarizing and hyperpolarizing current injections respectively. Figure panel demonstrates the parameters measured to assess the intrinsic electrical properites of human hippocampal neurons: input resistance of the neuronal membrane (Ri), AP frequency measured at depolarizing current injection of 1.5 times the rheobase, AP afterhyperpolarization (AHP), and sag. d-g. Input resistance (d), AP AHP (e), sag (f), and AP frequency (g) presented as box and whisker plots (median, lower/upper quartiles and min-max values). Statistical significance was tested with two-tailed Mann-Whitney test for input resistance, two-tailed unpaired t-test for AP frequency, and with two-tailed unpaired t-test with Welch’s correction for AHP, and. * p-value <0.05, ** p-value <0.01, ***p-value <0.001, ns – not significant. h. Firing frequency–current (F–I) curves showing action potential firing rates in response to increasing depolarizing current injections in human neurons in non-sclerotic (light red, left) and sclerotic (red, right) CA1. Colored traces represent individual neurons; the bold black line indicates the population average ± s.e.m., and the blue line depicts a linear fit to the average F–I curve. i. Each individual F–I curve was fitted with a line to calculate its slope. The distribution of F–I slopes across neurons presented as box and whisker plots (median, lower/upper quartiles and min-max values). Statistical significance was tested with two-tailed Mann-Whitney test. * p-value <0.05. Individual data points represent data from a single neuron. j. Representative traces of neuronal (non-sclerotic – light red, sclerotic – red) response to ZAP (chirp) protocol. k. The power spectrum derived from the chirp analysis showing the membrane impedance as a function of input frequency, reflecting the neuron’s frequency-dependent responsiveness. Traces show individual neurons (thin gray lines), population average (bold colored line), and ± s.e.m. (shaded area). l-m. Distribution of resonance frequency (f_res_; l) and 3 dB cutoff frequency (m) across neurons from each hippocampal sub-region presented as box and whisker plots (median, lower/upper quartiles and min-max values). Statistical significance was tested with two-tailed unpaired t-test with Welch’s correction for resonance frequency and with two-tailed unpaired t-test for 3 db cutoff frequency. *** p-value <0.001, ns – not significant.

**Figure 3:**
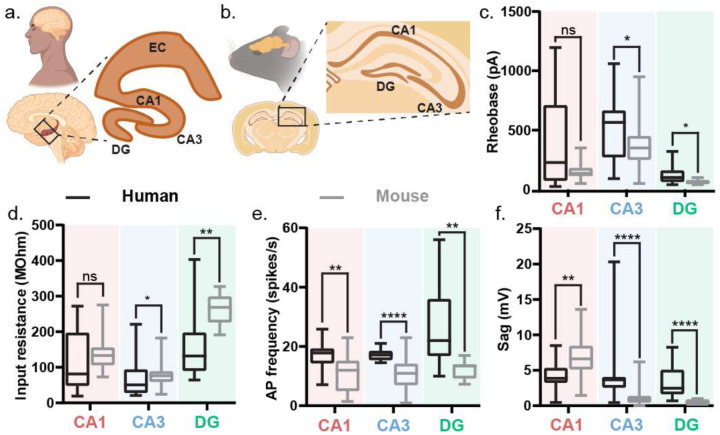
Human hippocampal neurons exhibit lower intrinsic excitability but higher action potential firing rates than mouse neurons. a. Schematic showing a sagittal section of a human brain with a box highlighting the hippocampus. Inset zooms in on the human hippocampus showing the different sub regions – dentate gyrus (DG), CA1, CA3, and entorhinal cortex (EC). b. Schematic showing a coronal section of a mouse brain with a box highlighting the hippocampus. Inset zooms in on the human hippocampus showing the different sub regions – DG, CA1, and CA3. c-f. Rheobase (c), input resistance (d), AP frequency (e), and sag (f) presented as box and whisker plots (median, lower/upper quartiles and min-max values). * p-value <0.05, ** p-value <0.01, **** p-value <0.0001, ns – not significant. For more details on statistics and exact p-values refer to Table 2.

**Figure 4:**
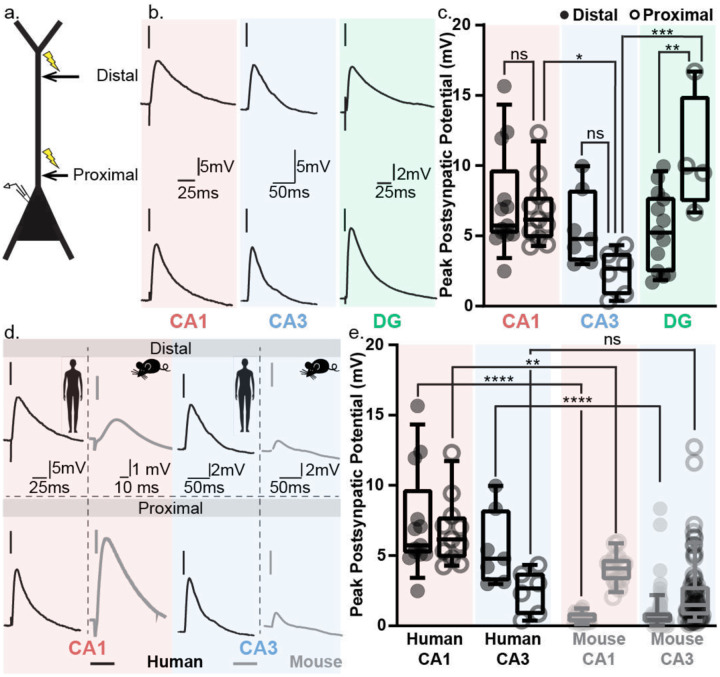
Human hippocampal neurons exhibit better long-range signal transmission compared to mouse neurons. a. Schematic of a neuron showing the experimental strategy. Hippocampal neuron was patch-clamped at the soma to record postsynaptic potentials (PSP) in response to the electrical stimulation of axonal inputs at distal versus proximal dendritic locations (indicated by yellow thunderbolts). b. Representative PSP traces from human neurons in CA1 (red box), CA3 (blue box), and DG (green box) in response to electrical stimulation of axonal inputs at distal versus proximal dendritic locations. c. Peak postsynaptic potentials from human neurons in CA1 (red box), CA3 (blue box), and DG (green box) in response to electrical stimulation of axonal inputs at distal (filled circles) versus proximal (open circles) dendritic locations. Fitting a full-model two-way ANOVA confirmed a significant effect of hippocampal region (p-value = 0.0029) but not of stimulation location (p-value = 0.54), and a significant interaction between the two factors (p-value = 0.0028). Our data suggest significant hippocampal region-specific differences in the peak PSP amplitudes but this effect is more pronounced for proximal stimulation location. Post hoc Sidak’s multiple-comparison test was used to assess significance of the differences between responses to distal and proximal stimulation at CA1 (p-value = 0.95), CA3 (p-value = 0.19) and DG (p-value = 0.0092). Post hoc Tukey’s multiple-comparison test was used to assess significance of the differences between responses to distal stimulation across all hippocampal regions (DG-CA1 p-value = 0.22; DG-CA3 p-value = 0.99; CA1-CA3 = 0.40) and to proximal stimulation across all hippocampal (DG-CA1 p-value = 0.07; DG-CA3 p-value = 0.003; CA1-CA3 = 0.02). d. Representative PSP traces from human (black) and mouse (gray) neurons in CA1 (red box) and CA3 (blue box) in response to electrical stimulation of axonal inputs at distal versus proximal dendritic locations. e. Peak postsynaptic potentials from human (black) and mouse (gray) neurons in CA1 (red box), and CA3 (blue box) in response to electrical stimulation of axonal inputs at distal (filled circles) versus proximal (open circles) dendritic locations. Statistical significance of the difference in peak PSP amplitudes across all groups was tested using three-way ANOVA with hippocampal region, stimulation location and species are the three factors. Species and hippocampal region contributed significantly to the variance but not the stimulation location. There was significant interaction between species and hippocampal region, species and stimulation location, and hippocampal region and stimulation location, but not all three factors together. Taken together are data suggest that the differences seen in the peak PSP amplitude are species and hippocampal region specific and the effect of stimulation location varies with species and hippocampal region tested. Post hoc Sidak’s multiple-comparison test was used to assess significance of the differences between human and mouse neurons across different hippocampal regions (human distal CA1 – mouse distal CA1 p-value <0.0001; human proximal CA1 – mouse proximal CA1 p-value = 0.005; human distal CA3 – mouse distal CA3 p-value <0.0001; human proximal CA3 – mouse proximal CA3 p-value = 0.9997).

**Figure 5:**
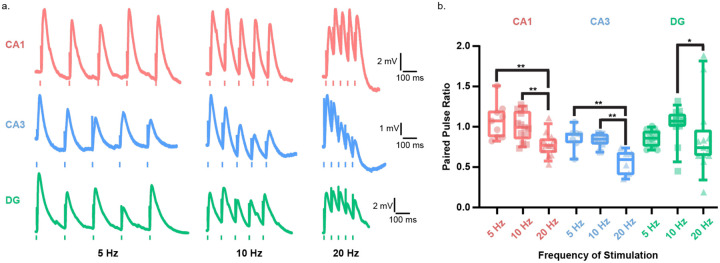
Human hippocampal neurons exhibit region-specific short-term plasticity a. Representative traces of postsynaptic response in human hippocampal neurons in CA1 (red), CA3 (blue), and DG (green) in response to train stimulation of distal inputs at frequencies of 5 Hz (left), 10 Hz (middle),and 20 Hz (right). b. Paired pulse ration (PPR) of the postsynaptic response to train stimulation at 5,10, and 20 Hz of distal inputs in CA1 (red), CA3 (blue), and DG (green) neurons presented as box and whisker plots (median, lower/upper quartiles and 10–90 percentiles). Statistical significance was tested with one-way ANOVA with Tukey’s post-hoc correction for multiple comparisons for CA1 and CA3, and with Kruskal-Wallis test with Dunn’s post-hoc correction for multiple comparisons for DG. * p-value <0.05, ** p-value <0.01.

**Figure 6:**
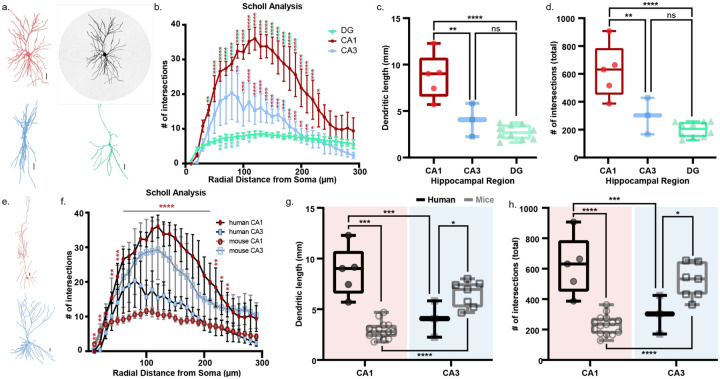
Human CA1 pyramidal neurons exhibit increased dendritic branching and complexity compared to their mouse counterparts. a. Representative reconstructions of human CA1 (red), CA3 (blue) and DG (green) neurons used for Scholl analysis. Scale bar – 25 μm. The grayscale CA1 neuron with concentric rings illustrates Scholl analysis implemented for assessing dendritic length and arborization. Each ring represents an incremental distance of 10 μm from the neuronal soma. Dendritic complexity is assessed in terms of the intersections the dendrites make with the rings at any given distance from the soma. b. Quantification of the number of dendritic intersections from 0–300 μm radial distances from the soma. Fitting a full-model two-way ANOVA confirmed a significant effect of differences between the hippocampal regions (p-value <0.0001), radial distance from the soma (p-value <0.0001), and significant interaction between the two factors (p-value <0.0001). Post hoc Tukey’s multiple-comparison test was used to assess significance of the differences between the groups. * p-value <0.05, ** p-value <0.01, *** p-value <0.001, **** p-value <0.0001. Significance between CA1-DG, CA1-CA3, and CA3-DG denoted by red-green, red-blue, and blue-green asterisks respectively. Only significant differences shown on the graph. c-d. Dendritic length (c) and dendritic complexity (d) depicted as number of intersections of human CA1 (red), CA3 (blue) and DG (green) neurons presented as box and whisker plots (median, lower/upper quartiles and min-max values). Statistical significance was tested with one-way ANOVA with Tukey’s post-hoc correction for multiple comparisons. ** p-value <0.01, **** p-value <0.0001, ns – not significant. e. Representative reconstructions of mouse CA1 (red) and CA3 (blue) neurons used for Scholl analysis. Scale bar – 25 μm. f. Quantification of the number of dendritic intersections from 0–300 μm radial distances from the soma. Statistical significance between human and mouse CA1 and CA3 was tested with multiple unpaired two-tailed t-tests followed by correction for multiple comparison using False Discovery Rate (FDR) test using the two-stage Benjamini, Krieger, and Yekutieli method. Only human and mouse CA1 showed significant differences at different radial distances from the soma as indicated by the asterisks. * p-value <0.05, ** p-value <0.01, *** p-value <0.001, **** p-value <0.0001. g. Dendritic length of human (black) and mouse (gray) CA1 and CA3 neurons presented as box and whisker plots (median, lower/upper quartiles and min-max values). Fitting a full-model two-way ANOVA confirmed a significant effect of differences between the species (p-value = 0.0092) but not hippocampal regions (p-value =0.38), and significant interaction between the two factors (p-value <0.0001). Our data suggest that human neuronal dendrites are significantly longer than their mouse counterparts but this effect is not the same across both the hippocampal regions. Post hoc Sidak’s multiple-comparison test was used to assess significance of the differences between the groups. * p-value <0.05, *** p-value <0.001, **** p-value <0.0001, ns – not significant. h. Dendritic complexity depicted as number of intersections of human (black) and mouse (gray) CA1 and CA3 neurons presented as box and whisker plots (median, lower/upper quartiles and min-max values). Fitting a full-model two-way ANOVA shows a significant interaction between the two factors –species and hippocampal region (p-value <0.0001), although neither species (p-value = 0.08) nor hippocampal region (p-value = 0.69) contributes significantly on its own to the effect seen. Our data suggest that the combination of hippocampal region and species specific differences significantly contributes to the observed difference. Post hoc Sidak’s multiple-comparison test was used to assess significance of the differences between the groups. * p-value <0.05, *** p-value <0.001, **** p-value <0.0001, ns – not significant.

Using intracellular whole cell patch-clamp recordings, high-resolution fluorescence microscopy, and morphological reconstructions, we present a comprehensive characterization of human and mouse principal neurons in hippocampal CA1, CA3, and DG. We compared their intrinsic electrical properties and excitability^45^ (Figure 1–3), synaptic strength (Figure 4) and plasticity (Figure 5) of distal and proximal dendritic inputs, and dendritic arborization (Figure 6). Combining the electrical signature and neuronal morphology, we confirmed neuronal locations (CA1, CA3, or DG), and identities (Figure 1d) to provide key insights into the synaptic, cellular and circuit architecture and function of principal neurons in the human hippocampus.

## Results

### Human Dentate Gyrus neurons are highly excitable compared to CA1 and CA3 neurons

**Extended Data Figure 2:**
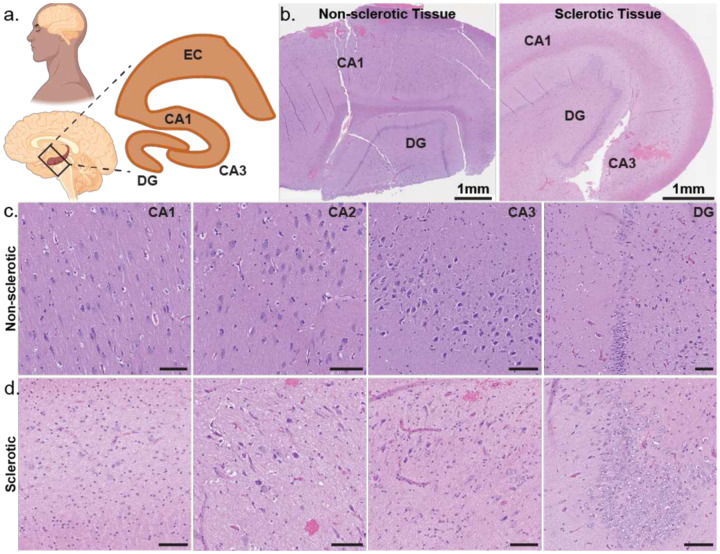
Comparison of sclerotic vs non-sclerotic human CA1 tissue samples a. Schematic showing a sagittal section of a human brain with a box highlighting the hippocampus. Inset zooms in on the human hippocampus showing the different sub regions – dentate gyrus (DG), CA1, CA3, and entorhinal cortex (EC). b. Low-power images of human hippocampus with hematoxylin-eosin stain to show different hippocampal regions in non-sclerotic (left) versus sclerotic (right) tissue. c-d. Magnified view of the hippocampal regions with hematoxylin-eosin stain highlighting normal neuronal architecture and population in non-sclerotic tissue (c) versus neuronal loss and aberrant cellular architecture in sclerotic tissue (d). Scale bar – 100 um.

**Extended Data Figure 3:**
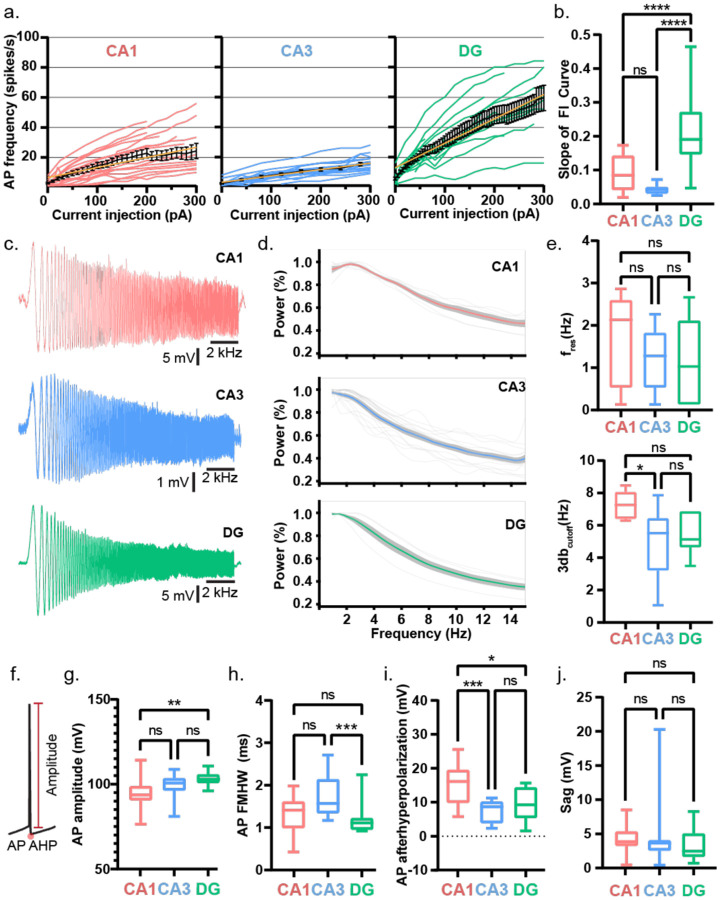
Intrinsic electrical properties of human hippocampal neurons a. Firing frequency–current (F–I) curves showing action potential firing rates in response to increasing depolarizing current injections in human CA1 (red), CA3 (blue), and DG (green) neurons. Colored traces represent individual neurons; the bold black line indicates the population mean ± s.e.m., and the yellow line depicts a linear fit to the average F–I curve. b. Each individual F–I curve was fitted with a line to calculate its slope. The distribution of F–I slopes across neurons presented as box and whisker plots (median, lower/upper quartiles and min-max values). Statistical significance was tested with one-way ANOVA with Tukey’s post-hoc correction for multiple comparisons. **** p-value <0.0001, ns – not significant. c. Representative traces of neuronal (CA1-red, CA3 – blue, and DG-green) response to ZAP (chirp) protocol where a sinusoidal current wave of 100pA peak-to-peak amplitude (−50:+50pA) with linear frequency increase from 0 to 15 Hz was injected into neurons over 30 s to assess their subthreshold impedance properties. d. The power spectrum derived from the chirp analysis showing the membrane impedance as a function of input frequency, reflecting the neuron’s frequency-dependent responsiveness. Traces show individual neurons (thin gray lines), population average (bold colored line), and ± s.e.m. (shaded area). e. Distribution of resonance frequency (f_res_; top) and 3 dB cutoff frequency (bottom) across neurons from each hippocampal sub-region presented as box and whisker plots (median, lower/upper quartiles and min-max values). Resonance frequency was defined as the frequency at which the normalized impedance peaked. The 3 dB cutoff was calculated as the frequency at which power dropped to 70.7% of the peak, reflecting the neuron’s effective frequency bandwidth. Statistical significance was tested with Kruskal-Wallis test with Dunn’s post-hoc correction for multiple comparisons for resonance frequency and with one-way ANOVA with Tukey’s post-hoc correction for multiple comparisons for 3 db cutoff frequency. * p-value <0.05, ns – not significant. f. Sample trace of a single action potential (AP) demonstrating amplitude and afterhyperpolarization (AHP) measurement. g-j. AP amplitude (a), half-width at full maxima (FMHW; b), AHP (c), and sag (d) presented as box and whisker plots (median, lower/upper quartiles and min-max values). Statistical significance was tested with one-way ANOVA with Tukey’s post-hoc correction for multiple comparisons for AP amplitude, and afterhyperpolarization, and with Kruskal-Wallis test with Dunn’s post-hoc correction for multiple comparisons for AP FMHW and sag. * p-value <0.05, ** p-value <0.01, *** p-value <0.001, ns – not significant.

First, we examined the intrinsic electrical properties of human neurons across CA1, CA3, and dentate gyrus (DG) regions (Figure 1). Among our 11 TLE patients, five exhibited varying degrees of hippocampal sclerosis or neuronal loss^6,22,46^ (Table 1). All CA3 tissue samples were non-sclerotic, all DG samples had mild neuronal dispersal; CA1 samples were derived from sclerotic and non-sclerotic tissue (Extended Data Figure 2). For the hippocampal interregional comparison, (Figure 1) we only included CA1 recordings from non-sclerotic tissue samples. We observed region-specific differences in action potential (AP) firing threshold, rheobase, input resistance, and AP firing frequency (Figure 1) of human hippocampal neurons. DG neurons had the lowest AP firing threshold (Figure 1e) and rheobase (Figure 1f), along with the highest input resistance (Figure 1g), indicating that they required the least current to reach threshold and were the most intrinsically excitable. In contrast, CA1 and CA3 neurons showed similar values for these parameters, suggesting comparable levels of intrinsic excitability between them. Consistently, AP firing frequency at 1.5× rheobase was similar in CA1 and CA3 neurons but significantly higher in DG neurons (Figure 1h). DG neurons exhibited a steeper F–I slope, reflecting a more rapid increase in firing rate with current injection compared to CA1 and CA3 neurons, which showed similar F–I slopes (Extended Data Fig. 3a–b). Next, we tested filtering properties. Chirp analysis revealed no difference in resonant frequency across regions. However, CA1 neurons exhibited the highest 3-dB cutoff, whereas CA3 and DG neurons showed similar, lower values (Extended Data Figure 3c–e). As predicted by the high input resistance, DG neurons exhibited the largest AP amplitudes (Extended Data Figure 3g) and the narrowest AP half-widths (Extended Data Figure 3h). CA1 neurons showed the most pronounced AP afterhyperpolarization (AHP) (Extended Data Figure 3i). Sag responses to hyperpolarizing current injections were similar across all three regions (Extended Data Figure 3j).

### Reduced Excitability and Filtering in Sclerotic CA1 Neurons

**Extended Data Figure 4:**
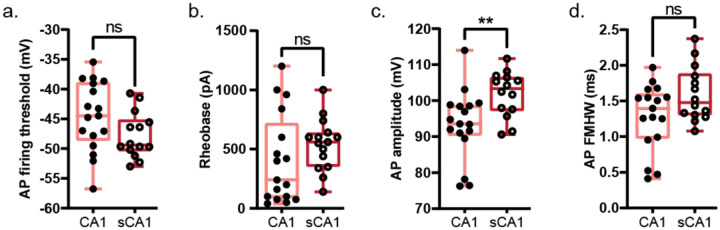
Intrinsic electrical properties of human sclerotic versus non-sclerotic CA1 neurons a-d. AP firing threshold (a), rheobase (b), AP amplitude (c), and AP half-width at full maxima (FMHW; d) presented as box and whisker plots (median, lower/upper quartiles and min-max values). Statistical significance was tested with two-tailed unpaired t-test for AP threshold, amplitude, and FMHW, and with two-tailed unpaired t-test with Welch’s correction for rheobase. ** p-value <0.01, ns – not significant. Individual data points represent data from a single neuron.

The availability of both sclerotic and non-sclerotic CA1 tissue enabled direct comparison of sclerotic pathophysiology (Figure 2). In sclerotic CA1, neurons showed lower input resistance (Figure 2d), but similar AP threshold (Extended Data Figure 4a) and rheobase (Extended Data Figure 4b). They exhibited reduced AHP (Figure 2e) and greater sag (Figure 2f) compared to non-sclerotic neurons. Firing rates were lower at 1.5× rheobase (Figure 2g), with shallower F–I slopes (Figure 2h–i). Chirp analysis revealed similar resonant frequencies, but a reduced 3-dB cutoff in sclerotic neurons (Figure 2j–m). AP amplitudes were larger (Extended Data Figure 4c), while kinetics remained unchanged (Extended Data Figure 4d). Given these differences, only non-sclerotic CA1 neurons were included in subsequent analyses of intrinsic electrical properties.

### Lower excitability but higher firing rates in human hippocampal neurons compared to mice

**Extended Data Figure 5:**
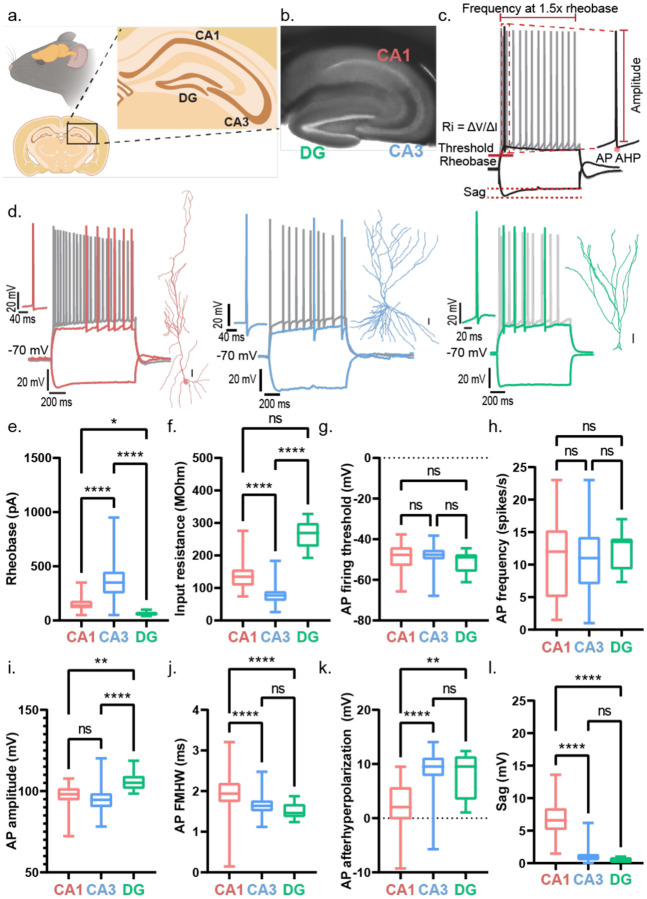
Intrinsic electrical properties of mouse hippocampal neurons a. Schematic showing a coronal section of a mouse brain with a box highlighting the hippocampus. Inset zooms in on the mouse hippocampus showing the different sub regions – dentate gyrus (DG), CA1, and CA3. b. Image shows a 400 μm thick acute mouse brain slice. Different hippocampal sub regions (CA1, CA3, and DG) demarcated within the slice from where electrophysiological recordings were obtained. c. Sample traces of fire and sag properties from a hippocampal neuron in response to depolarizing and hyperpolarizing current injections respectively. Figure panel demonstrates the parameters measured to assess the intrinsic excitability of human hippocampal neurons: rheobase (minimum current required to elicit action potential (AP)), input resistance of the neuronal membrane (Ri), AP firing threshold, AP frequency measured at depolarizing current injection of 1.5 times the rheobase, AP amplitude, and sag. d. Representative traces of fire and sag properties recorded from mouse CA1 (red), CA3 (blue), and DG (green) in response to depolarizing and hyperpolarizing current injections respectively. Colored firing trace in each panel corresponds to AP firing at rheobase, while the gray firing trace corresponds to AP firing at current injection 1.5 times the rheobase. Left inset shows a magnified view of a single AP. Right inset shows a reconstructed neuron that was filled with dye during electrophysiological patch-clamp recordings and subsequently stained and imaged. Scale bar – 25 μm. Fire and sag properties, and neuron morphological helped confirm the neuronal type recorded. e-l. Rheobase (e), input resistance (f), AP firing threshold (g) AP frequency (h), AP amplitude (i), AP full-width at half-maxima (FMHW; j), AP afterhyperpolarization, and sag (l) presented as box and whisker plots (median, lower/upper quartiles and min-max values). Statistical significance was tested with Kruskal-Wallis test with Dunn’s post-hoc correction for multiple comparisons for all parameters. * p-value <0.05, ** p-value <0.01, **** p-value <0.0001, ns – not significant.

Is the intrinsic electrophysiological signature of hippocampal neurons conserved across human and murine species? We compared the intrinsic electrical properties of human hippocampal cell-types with their counterparts in the wild-type mouse (Extended Data Figure 5), the most common model organism. Rodent studies have primarily informed the classical view of the hippocampal neurons and circuit. Our analysis of the intrinsic excitability of mouse hippocampal neurons across sub-regions aligns with the classical view^47–53^. Parameters such as rheobase (Extended Data Figure 5e) and input resistance (Extended Data Figure 5f) show that mouse DG neurons have the highest intrinsic excitability compared to CA1 and CA3 with similar AP firing threshold (Extended Data Figure 5g) and firing frequency (Extended Data Figure 5h). While CA1 is less excitable than DG neurons, CA3 neurons are least excitable among the three hippocampal sub-regions. DG neurons have the highest AP amplitudes while CA1 and CA3 have similar values (Extended Data Figure 5i). CA1 neurons have the slowest AP kinetics (half-width at full maxima) while CA3 and DG APs are similarly fast (Extended Data Figure 5j). CA1 neurons have the smallest AP afterhyperpolarization (Extended Data Figure 5k) and the biggest sag (Extended Data Figure 5l) while CA3 and DG have similar values.

**Extended Data Figure 6:**
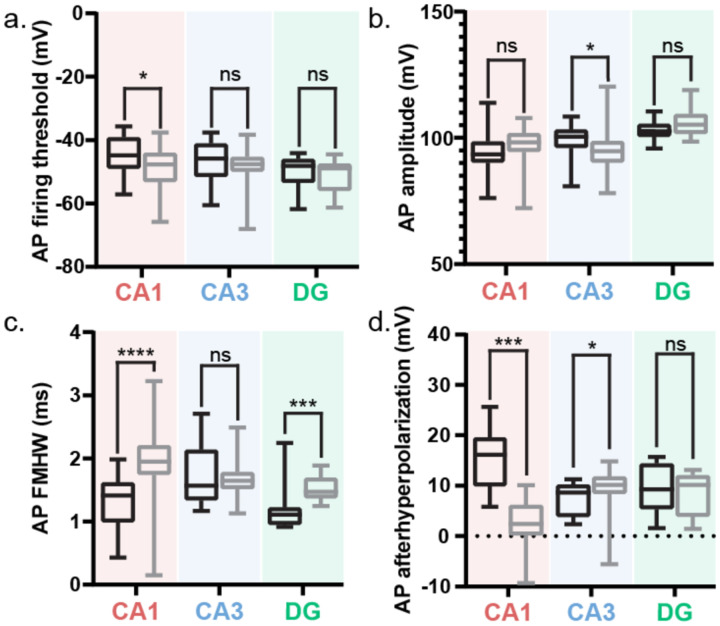
Intrinsic electrical properties of human versus mouse hippocampal neurons a-d. AP firing threshold (a), amplitude (b), half-width at full-maxima (FMHW; c), and afterhyperpolarization (d) presented as box and whisker plots (median, lower/upper quartiles and min-max values). * p-value <0.05, *** p-value <0.001, **** p-value <0.0001, ns – not significant. For more details on statistics and exact p-values refer to Table 2.

Direct comparison of membrane voltage changes to depolarizing and hyperpolarizing current injection steps in human and mouse hippocampal neurons (Figure 3, Table 2) revealed that human neurons have lower intrinsic excitability. Human CA3 and DG neurons had higher rheobase (Figure 3c) and lower input resistance (Figure 3d) than their mouse counterparts, while CA1 neurons in both species were comparable for these two parameters. However, the AP firing threshold was similar between the two species in CA3 and DG neurons, while human CA1 had a higher firing threshold than mouse CA1 neurons (Extended Data Figure 6a). Despite the lower (in CA3 and DG) or comparable (in CA1) intrinsic excitability, human neurons across all hippocampal sub-regions demonstrated a higher AP firing frequency than their mouse counterparts (Figure 3e). Human CA1 neurons had a smaller sag than mouse CA1 neurons, but human CA3 and DG had a larger sag than their mouse counterparts (Figure 3f). AP amplitudes were similar across species in CA1 and DG neurons but human CA3 neurons demonstrated slightly higher AP amplitude than mouse CA3 neurons (Extended Data Figure 6b). Human CA1 and DG neurons had narrower AP half widths than their mouse counterparts, while human and mouse CA3 neurons had similar kinetics (Extended Data Figure 6c). Human CA1 and CA3 neurons had larger AP afterhyperpolarization than their mouse counterparts while human and mouse DG neurons had similar AP afterhyperpolarization (Extended Data Figure 6d).

### Enhanced distal transmission in human hippocampal neurons

After defining species- and region-specific differences in intrinsic excitability, we examined how neuronal and network output vary across hippocampal sub-regions and between species. To do this, we electrically stimulated axonal inputs along the apical dendrites of hippocampal neurons and recorded somatic responses. In rodents, the canonical cortico-hippocampal circuit^16,54^ posits that long-range inputs from the entorhinal cortex target distal dendrites, while intra-hippocampal inputs (e.g., CA3 to CA1, DG to CA3) synapse more proximally. This input stratification supports diverse integration mechanisms such as spike-timing and input-timing dependent plasticity^55–57^.

This murine spatial segregation of inputs may not hold in humans. While the rodent hippocampus pyramidal layer is ~5 neurons thick, in humans it can span ~20 neuron strata^58^. Thus, inputs classified as “distal” in one neuron may be proximal to another, and vice versa. Therefore, we refer to stimulation sites relative to the soma—using “proximal” and “distal”—rather than intra- or extra-hippocampal.

The peak postsynaptic potentials (PSP) in response to distal and proximal stimulation were similar in CA1 and CA3 neurons (Figure 4b–c). In DG neurons, the response was higher to proximal than to distal stimulation. Across the hippocampal regions, the response to distal input stimulation was similar in all three regions, and the response to proximal input stimulation was lowest in CA3 neurons and similar in CA1 and DG neurons (Figure 4c).

Similar postsynaptic responses to stimulation of distal and proximal inputs in human CA1 and CA3 neurons contrasts starkly with mouse CA1 and CA3 neurons (Figure 4d–e, Table 3). In the mouse, postsynaptic responses at the soma to distal inputs are smaller than proximal input evoked responses (Figure 4d–e). Across mouse hippocampal regions, similar to human neurons, postsynaptic responses in CA1 and CA3 neurons to distal input stimulation were similar (Table 3). Mouse CA1 and CA3 neurons showed similar postsynaptic responses to proximal input stimulation, unlike the human neurons (Table 3). Postsynaptic responses to distal stimulation were higher in human CA1 and CA3 neurons than in mouse neurons (Figure 4e, Table 3). For proximal input stimulation, postsynaptic responses in human CA1 neurons were higher than mouse CA1 neurons but for CA3 neurons the postsynaptic responses across species were similar (Figure 4e, Table 3).

### Sub-region-specific short-term plasticity in human hippocampal neurons

To assess the short-term plasticity of synaptic inputs to distal dendrites of human hippocampal neurons, we electrically stimulated the axons with electrical pulse trains at three frequencies and recorded the postsynaptic responses from the soma (Figure 5, Table 4). The postsynaptic responses to distal input stimulation at human CA1 and DG neurons showed short-term potentiation (PPR >1) at 5 and 10 Hz, but short-term depression (PPR <1) at 20 Hz. In human CA3 neurons, the postsynaptic response to distal input stimulation showed short-term depression at all three frequencies (Figure 4b).

### Increased dendritic length and branching complexity in human hippocampal CA1

To examine neuronal morphology, each patch-clamped neuron was filled with biocytin during intracellular recordings, followed by post-hoc immunostaining, imaging, and reconstruction. We performed Scholl analysis to quantify dendritic length and complexity across hippocampal regions and species (Figure 6), using concentric rings centered on the neuronal soma at 10 μm intervals from the soma to measure dendritic branching calculated as the number of dendritic intersections in each ring.

In human neurons, dendritic intersections were similar across DG, CA1, and CA3 up to ~50 μm from the soma. Beyond this point, DG neurons exhibited minimal additional branching, whereas CA1 and CA3 neurons showed more extensive arborization. Human CA3 neurons branched earlier, peaking at ~80 μm, while CA1 neurons reached a higher peak at ~120 μm (Figure 6b). Among the three regions, human CA1 neurons had the longest dendrites (Figure 6c) and the most complex arborization (Figure 6d).

Mouse CA3 neurons showed greater branching than mouse CA1 neurons, as reported^59^. While CA3 branching was similar across species, human CA1 neurons exhibited more branching than mouse CA1 neurons (Figures 6f). Human CA1 neurons have longer dendrites and more complex dendritic arbors than the mouse CA1 neurons. This pattern is reversed in CA3, where mouse neurons are more complex than human ones (Figure 6g–h). These results highlight species- and sub-region-specific morphological differences: human CA1 neurons exhibit extensive and complex dendritic trees that may enhance integrative and computational capacities.

## Discussion

Our study reveals distinct region-, pathology-, and species-dependent differences across hippocampal neuron types, revising current models of human hippocampal function in health and disease. High-resolution characterization of human hippocampal single neuron intrinsic properties, synaptic physiology, and morphology is essential to understand the human hippocampal circuit, which normally supports key brain functions such as episodic memory and spatial navigation, and in pathological states, can impair these functions and cause TLE. This information remains poorly defined due to rare access to live human tissue with intact integrity of the cortico-hippocampal circuit. We established a unique collaborative setting that enabled rapid transport and preparation of acute slices from en-bloc resected human hippocampi for long-term, high-quality recordings. We systematically characterized the intrinsic excitability, synaptic output, short-term plasticity, and dendritic morphology of CA1, CA3, and DG neurons to generate a comprehensive dataset that lends insight into the function of human hippocampal neuron types.

Evolutionary differences across species may impact the translation of interventions to treat cognitive, psychiatric, and epilepsy disorders affecting the hippocampus. We found that DG granule cells are the most excitable hippocampal neuron type across species. However, significantly lower sag in CA1 neurons in human versus mouse points to evolutionary divergence in hyperpolarization-activated cyclic nucleotide-gated (HCN) channels^60,61^. Consistent with this, we find that in human CA1 pyramidal neurons, synaptic stimulation of distal dendrites elicits somatic responses comparable to proximal inputs—contrasting sharply with the strong attenuation of distal signals in mice^62,63^. Together with their complex and unique dendritic arborization, these findings suggest that human CA1 pyramidal neurons have evolved structurally and functionally to support enhanced dendrite-to-soma signal propagation and synaptic plasticity to integrate multimodal sensory and cognitive inputs. Direct comparison of pathologically defined sclerotic versus non-sclerotic human CA1 neurons revealed reduced excitability, diminished AHP, and impaired high-frequency signal propagation in sclerotic tissue. Our results directly correlating electrophysiology with pathology in human TLE patient tissue contrast with mixed reports on changes in HCN channel expression and sag, as well as increased intrinsic excitability and AHP in mouse models of TLE^64–68^, and emphasize the importance of species-specific pathophysiology. By directly defining the cellular dynamics of human hippocampal neurons, our study highlights how patient-derived tissue provides unique insights into pathological mechanisms and offers a foundation to develop cell-type-specific therapeutic strategies for epilepsy and other neurological and psychiatric disorders.

### Regional differences in intrinsic excitability across species

Our inter-regional analysis showed that DG neurons were the most excitable among human hippocampal sub-regions, with the lowest AP threshold and rheobase, highest input resistance, steepest F–I slopes, and greatest steady-state firing frequency. In contrast, CA1 and CA3 neurons exhibited similar excitability. This differs from mice, where excitability follows the sequence DG > CA1 > CA3. Thus, while DG neurons are consistently the most excitable across species, CA1 and CA3 converge in humans, raising the possibility that human CA3 is intrinsically more excitable than in mice, or that human CA1 is relatively less so.

This divergence extended to AHP and sag. In humans, CA1 neurons showed the smallest AHP, whereas in mice they showed the largest. Direct comparisons revealed that human CA1 had larger AHP than mouse CA1, while CA3 and DG were lower and similar, respectively, across species. This suggests that the relative ability of CA1 neurons to repolarize after an AP differs between humans and mice. Sag, which depends on HCN channel conductance, also varied across species. In humans, sag was comparable across all three hippocampal subregions, whereas in mice it was strongest in CA1 and similar in CA3 and DG. These findings point to species-specific expression or distribution of HCN and other ionic channels and highlight fundamental differences in how hippocampal neurons regulate excitability.

Human CA3 and DG neurons had lower intrinsic excitability than their mouse counterparts, with higher rheobase and lower input resistance. CA1 excitability was comparable, although human CA1 displayed a higher AP threshold. These features likely reflect differences in cellular morphology: larger human neurons, with greater membrane surface area, have more leak conductance and ion channels, lowering input resistance and raising rheobase. Notably, despite reduced intrinsic excitability, human neurons fired at higher frequencies than mouse neurons across all sub-regions. This paradox suggests that additional factors—such as channel kinetics, dendritic integration, or morphological specializations—enhance firing output in humans.

### Effects of hippocampal sclerosis on CA1 excitability

The availability of both sclerotic and non-sclerotic CA1 tissue enabled us to directly assess pathology-dependent alterations in excitability. Sclerotic CA1 neurons exhibited reduced excitability, lower firing rates, and shallower F–I slopes, accompanied by decreased AHP and increased sag. This contrasts with higher intrinsic excitability, firing rate, and lower AHP reported in mouse models of TLE^64^, suggesting species- or pathology-specific mechanisms in human epilepsy. Chirp analysis revealed a lower 3-dB cutoff in sclerotic CA1 neurons, indicating impaired frequency responsiveness and diminished capacity for high-frequency signal propagation. Functionally, these changes suggest that sclerosis imposes a low-pass filter on CA1 neurons, reducing their ability to process fast inputs, disrupting temporal coding^42,69^ and impacting downstream excitability^70^.

The combined alterations in AHP, sag, and 3-dB cutoff point to dysregulation of K^+^ and HCN channel conductances^60,61,64,71–73^. Reduced AHP may weaken frequency adaptation, allowing neurons to remain more excitable during sustained input, while increased sag may promote rebound excitability. Together, these changes could paradoxically support both impaired cognitive processing—by narrowing the bandwidth for information transfer—and enhanced vulnerability to hypersynchronous activity, facilitating seizure propagation. Thus, sclerosis in human CA1 neurons appears to simultaneously compromise normal signal processing and predispose circuits to pathological hyperexcitability. Alternatively, the lower input resistance, shallower F-I slopes, and reduced 3-dB cutoff observed in human sclerotic neurons could stem from higher leak conductances in sclerotic neurons, as a protective mechanism evolved in humans against the increased excitability observed at seizure focus^74^. These findings underscore the marked divergence between rodent TLE models^64,65^ and human TLE, highlighting the need for caution when extrapolating from mouse to human, as key mechanisms of abnormal excitability may be differ fundamentally across species.

### Enhanced dendritic signal preservation in human neurons

Examining somatic responses to proximal and distal dendritic stimulation revealed striking species differences. In human hippocampal neurons, distal inputs evoked somatic responses that were comparable in strength to those from proximal inputs in both CA1 and CA3. In contrast, mouse neurons show strong attenuation of distal inputs^62,63^, a phenomenon attributed to dendritic cable properties, gradients of leak conductances (e.g., HCN and potassium channels) along the dendrite^75,76^, and morphological constraints. Human neurons, despite having substantially longer dendrites, preserved distal signals far more effectively—likely due to structural specializations, differences in ion channel distribution^66,77^, enhanced dendritic excitability^4,78^. Alternatively, stronger distal responses in humans may reflect greater synaptic drive, higher spine density, or increased receptor expression. Definitive testing of these possibilities will require direct dendritic recordings to quantify input strength at proximal versus distal sites, as well as quantifying HCN channel expression in human hippocampal neurons.

Morphology may contribute to differences in signal preservation. In mice, CA2 neurons exhibit stronger somatic responses to distal inputs compared to CA1 neurons—a property linked to their dendritic architecture^79^. CA2 neurons have more distal branches, fewer obliques, and primary dendrites that converge closer to the soma, facilitating summation. These morphological features are shared by human CA1 neurons. CA1 dendrites display more extensive branching in humans vs. mice (Figure 6; Mertens et al^7^.) and human neurons exhibit branch geometries facilitating signal convergence near the soma—potentially supporting efficient integration of distal inputs.

### Sub-region-specific synaptic plasticity

Short-term plasticity analysis demonstrated region- and frequency-specific differences in humans. Distal inputs onto CA1 and DG neurons facilitated at 5–10 Hz but depressed at 20 Hz, whereas CA3 distal inputs consistently depressed across frequencies. In contrast, in mice, distal inputs from the entorhinal cortex—temporo-ammonic (TA) inputs to CA1 and lateral perforant path inputs to DG—show facilitation across all frequencies^80–83^.

At 20 Hz stimulation—a frequency shown to interrupt seizure activity in rodents^84,85^—all human hippocampal sub-regions showed synaptic depression. This frequency-dependent depression may reflect a synaptic mechanism that could be implemented for deep-brain stimulation protocols to suppress seizure propagation.

Together, these findings highlight both conserved and divergent principles of hippocampal organization across species. While DG consistently emerges as the most excitable region, CA1 and CA3 differ in relative excitability and synaptic properties between humans and mice. Such differences in intrinsic excitability, enhanced long-range signal transmission, and a more complex dendritic morphology challenge rodent-based conventions, underscoring the importance of direct human studies. By elucidating species-specific principles of hippocampal function, our work highlights the limitations of extrapolating from animal models and reinforces the need for human studies to inform targeted therapies^86^. Future work combining dendritic recordings, ion channel profiling, microcircuit dissection^87^ and computational modeling will be essential to link the observed electrophysiological and morphological features to human cognition and disease vulnerability.

## Materials and Methods

### Human patient tissue samples

Human tissue samples were obtained with informed patient consent from 11 individuals with TLE. The tissue collection and associated experiments were reviewed and approved by the NYU Langone Health Institutional Review Board (IRB). Informed consent was obtained from all patients in accordance with the IRB. Tissue samples were deidentified, such that patient identity was only available to clinicians. Samples included both hippocampal hemispheres and patients of both sexes, with an age range of 12 to 48 years (Table 1).

### Animals

All experiments were conducted in accordance with the National Institutes of Health guidelines and with the approval of the *New York University Grossman School of Medicine Institutional Animal Care and Use Committee (IACUC)*. Adult mice aged 12–14 weeks of either sex with genetic background of C57BL/6 were used for the experiments. Animals were housed in rooms with 12:12 light dark cycle, temperature maintained at 70–72 degrees Fahrenheit (21–22 degrees Celsius) and humidity at 30–70%. Littermates were co-housed up to 5 mice per cage and had ad libitum access to food and water.

### Human patient sample acquisition

All patients underwent epilepsy surgical workups that included detailed history and neurological examination, long term video EEG monitoring, brain MRI, neuro-psychological evaluation, and often also included FDG-PET and an intra-carotid amytol study (ICA or Wada test.) Intracranial electrode implants for invasive EEG monitoring were often necessary to select appropriate surgical resection candidates. These tests defined the seizure onset zone which determined the anatomical resection targets, selecting appropriate patients for targeted unilateral resection of neocortical and hippocampal tissue for inclusion in this study. The hippocampus and parahippocampal gyrus were micro-surgically harvested in a manner that minimized mechanical and hypoxic tissue injury^88,89^. Resected tissue samples were transported in 50 ml Falcon tubes filled with transport solution continuously bubbled with carbogen gas (95% O_2_, 5% CO_2_) from a balloon reservoir (Extended Data Figure 1). The transport apparatus was kept on ice throughout transit to preserve tissue viability. A substantial portion of resected tissue was sent to the neuropathological laboratory for complete histological assessment, neuropathological diagnosis, and sub-classification according to international league against epilepsy (ILAE) sclerosis standards (see Table 1). Upon clearance from Neuropathology, the tissue was transported to the laboratory for experimentation. From the tissue collection in the surgery room to arrival in the lab for slice preparation, it took approximately 10–15 minutes.

### Slice preparation and Electrophysiology

#### Preparation of human tissue slices

Tissue blocks of human hippocampus (typically ~1 cm^3^) were removed from the brain, and immediately submerged in ice-cold NMDG-based dissection artificial cerebrospinal fluid (dACSF) containing (in mM): NMDG 93, KCl 2.5, NaH_2_PO_4_ 1.25, NaHCO_3_ 30, HEPES 20, glucose 25, thiourea 2, Na-ascorbate 5, Na-pyruvate 3, CaCl_2_ 0.5, MgCl_2_ 10 with pH adjusted to 7.3, and saturated with continuous carbogen gas bubbling. Hippocampal tissue blocks were trimmed to best preserve the structure of the parahippocampal gyrus. Specimen blocks were then glued (cyanoacrylate glue; Loctite 401, Henkel) to the stage of a VT 1200S vibratome (Leica microsystems, Wetzlar, Germany) and submerged ice-cold dACSF saturated with carbogen bubbling. For sectioning, the blade was positioned about 1 mm from the dorsal edge of the brain and sections were cut at a blade feed rate of 0.12 mm/s with an amplitude of 1.00 mm. Slices were incubated for 30 minutes in artificial cerebrospinal fluid (ACSF) containing (in mM): NaCl 125.0, glucose 22.5, NaHCO_3_ 25.0, KCl 2.5, NaH_2_PO_4_ 1.25, sodium pyruvate 3.0, ascorbic acid 1.0, CaCl_2_ 2.0, MgCl_2_ 1.0, with pH adjusted to 7.3, osmolarity of ~310 mOsm/l, and saturated with 95% O_2_ and 5% CO_2_. The ACSF was maintained at 35°C for the 30-minute incubation and then kept at room temperature (22–24°C) until recording. All the chemicals were procured from Sigma Aldrich.

#### Preparation of mouse tissue slices

Slices were prepared as described previously^55–57,90^. Briefly, adult mice (12 – 14 week old) were deeply anesthetized with 5% isoflurane and transcardially perfused with ice-cold dACSF. After decapitation, the brain was dissected out and quickly immersed in ice-cold dACSF. The olfactory bulbs and the cerebellum were separated from the brain block by two coronal cuts followed by a midsagittal cut to separate the two hemispheres. Using a brain-block, the two hemispheres were cut ventro-medially at a 10° angle. The two brain blocks were then glued to the vibratome stage such that the ventral side was glued on, the medial side was facing the blade, and the dorsal side was facing upwards, submerged in ice-cold dACSF. The brain was then sectioned as described above for the human tissue slices.

#### Electrophysiology

Patch-clamp recordings were made from pyramidal neurons (PNs) in CA1 and CA3, and granule cells (GC) in DG using a MultiClamp 700B amplifier (Axon Instruments) controlled by the pClamp 9 software (Molecular Devices), and junior micromanipulators on movable motorized shifting tables (Luigs & Neumann). Sampling interval and filter settings were 50 μs and 10 kHz respectively. Cells were visualized by differential interference contrast (DIC) microscopy through a 60x water-immersion objective (NA 1.0; Olympus) using an Olympus BX51 microscope and a Hamamatsu ORCA-R2 CCD camera using ImageJ Micromanager imaging acquisition software. All experiments were conducted at a temperature of 33–35°C, maintained by constant superfusion (flow rate 3–4 ml/min) of ACSF, heated by an inline solution heater (SH-27B with TC-324B controller; Warner Instruments, Hamden, CT, USA) and monitored by a thermistor placed between the inflow site and the slice in the recording chamber.

Patch pipettes were pulled with P-1000 micropipette puller (Sutter Instruments Co., Novato, CA, USA) from borosilicate glass capillaries with filament (1.5 mm O.D. × 10 cm length × .86 mm I.D., Sutter Instruments BF150-86-10). Open tip pipette resistance was 3–4 MΩ when filled with intracellular solution containing (in mM): KMeSO_4_ 135, KCl 5, EGTA 0.1, HEPES 10, NaCl 2, Mg-ATP 5, Na_2_-GTP 0.4, Na_2_Phosphocreatine 10, and biocytin (4 mg/mL; 0.2%; Invitrogen) with a pH of 7.35 and an osmolarity of 300 mOsm/l. Current clamp recordings were obtained with access resistances up to 20 MΩ, compensated in bridge balance mode. The cells were maintained at a resting membrane potential of −70 mV by current injection to allow for the comparison of amplitudes of post-synaptic potentials (PSPs). All the chemicals were procured from Sigma Aldrich unless stated otherwise.

Post-synaptic responses to electrical stimulation of input fibers were evoked with constant current stimulation units (Digitimer Ltd.) delivering 0.1 ms long 25–200 μA current pulses through monopolar electrodes (ACSF-filled pipettes) mounted on manual micromanipulators (Siskiyou). In human slices, while recording evoked responses in neuronal somata, proximal stimulation electrodes were placed 2–3 cell diameters above the recording site while for distal stimulation the stimulating electrode was placed close to the edge of the gray matter layer in the slice. In mouse slices, for proximal stimulation in CA1 and CA3 the stimulating electrode was placed in *stratum radiatum* (SR) and for distal stimulation in *stratum lacunosum-moleculare* (SLM).

All the electrophysiology data were analyzed, and graphs were generated using IgorPro 8.0.4.2 (Wavemetrics) using custom written scripts.

### Immunohistochemistry

#### Tissue preparation

Human brain tissue from epilepsy patients and wild-type mouse brain tissue slices were drop-fixed in 4% PFA overnight, and washed and stored in PBS at 4°C. To begin tissue clearing, the slices were thoroughly washed with 0.1M PB. Then the tissues were placed in CUBIC#1 clearing solution for two days at room temperature. Before starting the immunostaining protocol, the slices were washed thoroughly with 0.1M PB. Slices were permeabilized with 0.5% PBS-Triton X (PBST), blocked in 3% Goat serum in PBS with 0.2% Triton for 4 hours in room temperature, and incubated overnight in 4°C with primary antibodies in 3% Goat Serum in 0.2% PBST. Slices were thoroughly washed with 0.2% PBST and then incubated overnight in 4°C with secondary antibodies in 3% Goat Serum in 0.2% PBST. Slices were washed thoroughly with 0.1M PB, and then placed in CUBIC#2 clearing solution for 1 hour. Slices were mounted on glass slides in CUBIC#2 solution.

#### Imaging

For imaging, light sheet theta microscope^91,92^ (implemented as ClearScope, MBF Biosciences) was utilized to image large volumes of human tissue. This highly optimized light sheet microscopy approach allowed for high-speed imaging of very large, cleared samples (compatible with multiple clearing methods) at high resolution (5x/0.28NA and 20x/1.0NA objectives) for imaging possibilities at multiple resolutions, as needed. The resulting datasets (TB scale) were stitched by a custom image stitching pipeline (based on Terastitcher) and downloaded at multiple resolutions to allow smooth visualization of the dataset. For imaging mouse neurons, a confocal microscope (20x/0.8NA) was utilized.

### Neuron Reconstruction

Using the stacked images, the neurons were three-dimensionally reconstructed using Neurolucida 360 software from MBF Biosciences by tracing the soma, dendrites and axons. Then a Neurolucida software extension Neurolucida Explorer was used to export the 3D tracing of the neuron into numeric values, which provides information including the branch number, total dendritic length, total number of segments, area of cell body and other parameters. For this research, Scholl analysis method was used to quantify branching density of the neurons. This method conceptually draws concentric circles around the cell body at incrementally increasing diameter, starting at a radius of 10 μm, and counts the intersecting numbers of branches and terminal point along with the length of the dendrites.

## Extended Data

**Extended Data Figure 7:**
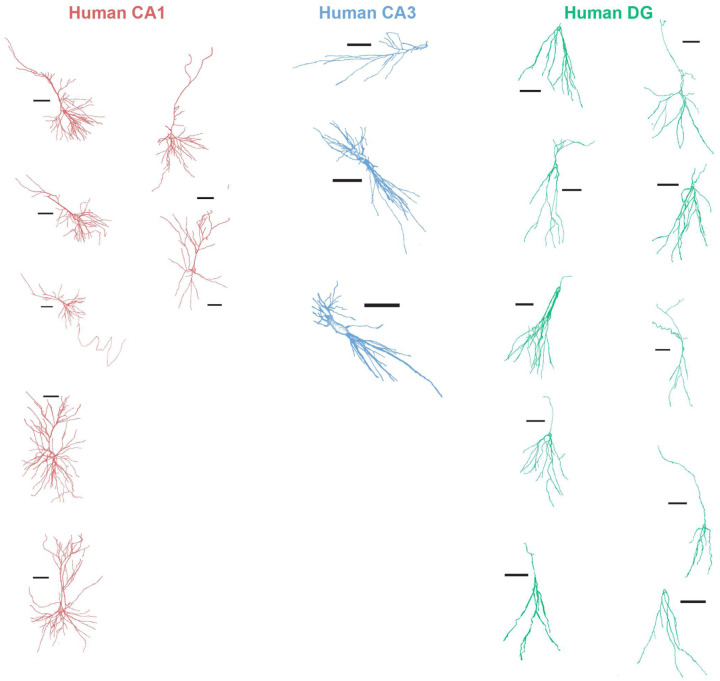
Human Neuronal Reconstructions Montage of neuronnal reconstructions of human CA1 (red), CA3 (blue) and DG (green) neurons. Scale bar – 100 um.

**Extended Data Figure 8:**
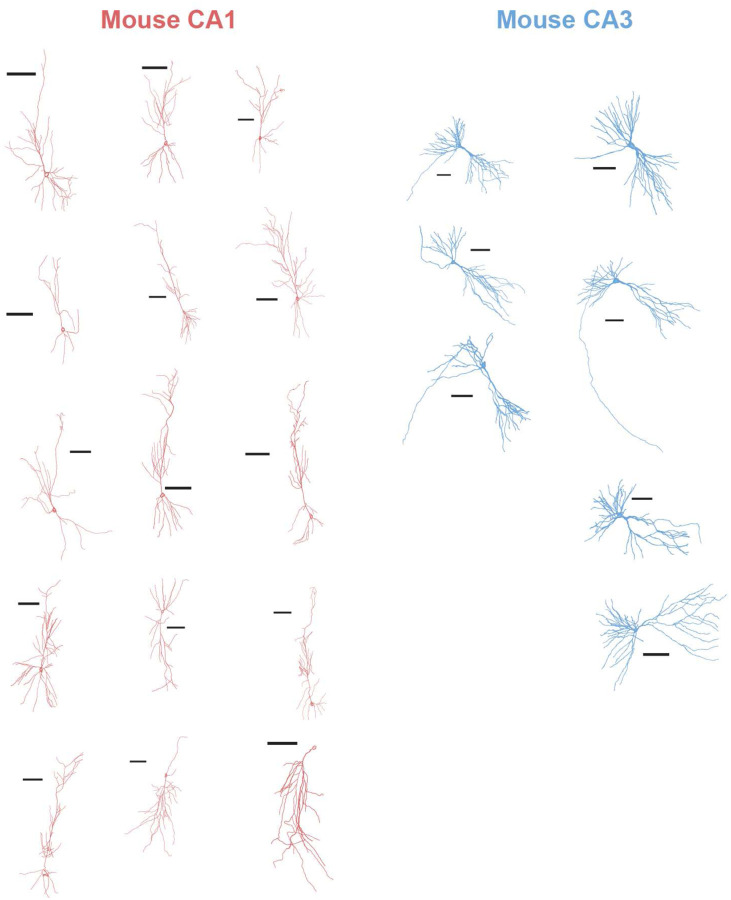
Mouse Neuronal Reconstructions Montage of neuronnal reconstructions of human CA1 (red) and CA3 (blue) neurons. Scale bar – 100 um.

## Supplementary Material

Supplementary Files

This is a list of supplementary files associated with this preprint. Click to download.

- SupplementalInformationTables.pdf
